# Evaluation of *Streptococcus mutans* strains possessing genes encoding collagen-binding proteins in the Japanese population

**DOI:** 10.1186/s12903-025-07276-5

**Published:** 2025-11-25

**Authors:** Makoto Okuda, Yuto Suehiro, Jinthana Lapirattanakul, Shuhei Naka, Michiyo Matsumoto-Nakano, Ryota Nomura, Rena Okawa, Kazuhiko Nakano

**Affiliations:** 1 Department of Pediatric Dentistry, Graduate School of Dentistry, The University of Osaka, Suita, Osaka Japan; 2 Department of Oral Microbiology, Faculty of Dentistry, Mahidol University, 6 Yothi Street, Rajthevi, Bangkok, Thailand 10400; 3 Department of Pediatric Dentistry, Okayama University Graduate School of Medicine, Dentistry and Pharmaceutical Sciences, Okayama, Okayama, Japan; 4 Department of Pediatric Dentistry, Graduate School of Biomedical and Health Sciences, Hiroshima University, Hiroshima, Hiroshima, Japan

**Keywords:** Collagen-binding protein gene, *cnm* gene, *cbm* gene, Japan, Multilocus sequence typing, Serotype, *Streptococcus mutans*

## Abstract

**Background:**

*Streptococcus mutans* harbors collagen-binding protein genes, namely *cnm* and *cbm*, which are implicated in its virulence and pathogenicity in both oral and extraoral infections. Although both genes were initially identified in *S. mutans* isolated from Japanese populations, their geographical prevalence, distribution, and genetic relatedness within Japan remain largely unexplored. This study investigates the prevalence of *S. mutans* strains carrying *cnm* and *cbm* genes across Japan, correlates these findings with clinical data, and analyzes the genetic relatedness of *cnm*-positive and *cnm*-negative strains using multilocus sequence typing (MLST).

**Methods:**

Dental plaque specimens were collected from 1248 individuals from eight Japanese cities (Hiroshima, Fukuoka, Nagasaki, Niigata, Okayama, Osaka, Tokushima, and Tokyo) and plated on selective medium for *S. mutans* isolation. *S. mutans* was confirmed in 523 subjects by colony morphology and PCR using species-specific primers, and the presence of the *cnm* and *cbm* genes was determined by PCR with gene-specific primers. Demographic (age, sex) and oral examination (caries prevalence, caries experience, number of teeth) data were recorded. MLST was employed to genotype selected *cnm*-positive and *cnm*-negative *S. mutans* strains to assess their clonal relationships.

**Results:**

Among 523 subjects possessing *S. mutans* (aged 3–90 years), we detected *cnm*-positive strains in all cities; specifically, the prevalence ranged from 5.5% in Okayama to 25.0% in Tokushima. In contrast, *cbm*-positive strains were less common and undetectable in some regions. Furthermore, subjects harboring *cnm*-positive *S. mutans* were significantly older (*p* = 0.002) and had higher caries prevalence and experience (*p* < 0.001). MLST revealed evolutionary relationships among *cnm*-positive strains across the cities but no discernible region-specific clustering. Clonal relationships partially reflected *cnm* gene distribution, particularly for exclusively *cnm*-positive or *cnm*-negative clonal complexes, but inconsistencies involving serotypes and *cnm* presence within some clonal complexes and sequence types were also noted.

**Conclusions:**

The *cnm*-positive *S. mutans* strains are widely distributed throughout Japan and are associated with increased age and caries burden. Although core genome analysis revealed some clonal patterns, the non-uniform distribution of the non-core *cnm* gene is likely influenced by horizontal gene transfer, providing *S. mutans* with adaptive advantages irrespective of its core genetic background or serotype.

**Supplementary Information:**

The online version contains supplementary material available at 10.1186/s12903-025-07276-5.

## Background

Dental caries is a dynamic, biofilm-mediated, and diet-modulated disease characterized by the net demineralization of dental hard tissues. It is multifactorial, driven by complex interactions among dental plaque microbiota, dietary habits, and a range of biological, behavioral, psychosocial, and environmental factors [1]. *Streptococcus mutans* is one of the oral microbes potentially associated with the development of dental caries based on its acidogenic and aciduric properties, and its ability to form robust biofilms in the presence of dietary sugars [2]. Once carious lesions reach dentin, collagen-binding proteins on the surface of *S. mutans* confer additional binding to the collagen-rich part of the tooth [3]. Surface proteins such as the surface protein antigen (PA, PAc, or SpaP) and the cell-wall associated protein A (WapA), which are ubiquitous in *S. mutans*, have been shown to bind collagen in vitro [4–6]; however, the clinical significance of these interactions remains unclear. A subset of *S. mutans* strains harboring the *cnm* and *cbm* genes, encoding collagen-binding proteins, showed significantly increased adhesion to collagen compared with strains lacking these two genes [7, 8].

The *cnm* and *cbm* genes were first identified in *S. mutans* strains isolated from the Japanese population [9–11]. In 2004, the 120-kDa Cnm protein, encoded by the *cnm* gene, was found to be responsible for the cold-agglutination phenotype of *S. mutans*, and subsequently, its ability to bind collagen and laminin was reported [9, 10]. Eight years later, the *cbm* gene encoding the Cbm protein was identified in clinical isolates of *S. mutans* that did not possess the *cnm* gene but exhibited strong collagen-binding activity [11]. The 3ʹ end of the *cbm* gene is flanked by the same core genes as the *cnm* gene [12], and the putative amino acid sequence of the collagen-binding domain of the Cbm protein is highly homologous to that of the Cnm protein [11]. Numerous studies have indicated the involvement of *S. mutans* strains harboring the *cnm* or *cbm* gene in diseases not only within an oral health context but also in systemic conditions. For example, when dentin caries reaches the pulp, causing pulpitis and endodontic infections, *S. mutans* possessing either the *cnm* or *cbm* gene has been detected at meaningful rates in inflamed pulp and infected root canal specimens. This suggests its potential as a source of bacteremia of dental origin, which is the initial step in the pathogenesis of systemic diseases induced by oral bacteria [13–15]. *S. mutans* harboring these collagen-binding genes can adhere to and invade human dental pulp fibroblasts and human coronary artery endothelial cells, facilitating their persistence and spread throughout the body [13, 14, 16]. In the *Galleria mellonella* (greater wax worm) infection model for systemic diseases, *S. mutans* strains with the *cnm* or *cbm* gene showed significantly greater virulence, evidenced by higher mortality rates, than those without these genes [14, 16]. Recent research has indicated the potential involvement of *S. mutans* strains harboring the *cnm* or *cbm* gene in the pathogenesis of various systemic complications, such as infective endocarditis, cardiovascular diseases, and IgA nephropathy [17–21]. The prevalence of *S. mutans* with these two collagen-binding protein genes was also associated with non-serotype *c*, the minor serotypes of *S. mutans* in oral cavities, and frequently detected from cardiovascular specimens of Japanese patients [22–24]. Recognizing this connection raises concerns about the link between oral health and overall systemic health from both the public and the scientific community in Japan [25–27].

Given the limited understanding of the geographical prevalence, distribution, and genetic relatedness of *S. mutans* carrying collagen-binding adhesins across Japan, this study aimed to survey *S. mutans* strains harboring the *cnm* and *cbm* genes among individuals from multiple regions of Japan, correlating these findings with their demographic data, including age and sex, as well as information from oral examination. Additionally, we sought to analyze the genetic relatedness of these *S. mutans* isolates, distinguishing between those possessing and those lacking the collagen-binding protein genes by multilocus sequence typing (MLST). This molecular typing method, based on the nucleotide sequences of multiple housekeeping genes, has successfully revealed clonal variations within the *S. mutans* population [28]. The purpose of this analysis was to determine whether regional specificity exists among *S. mutans* strains carrying collagen-binding genes in Japan and to elucidate the relationship defined by MLST based on the presence of collagen-binding genes.

## Materials and methods

### Subject information

**Fig. 1:**
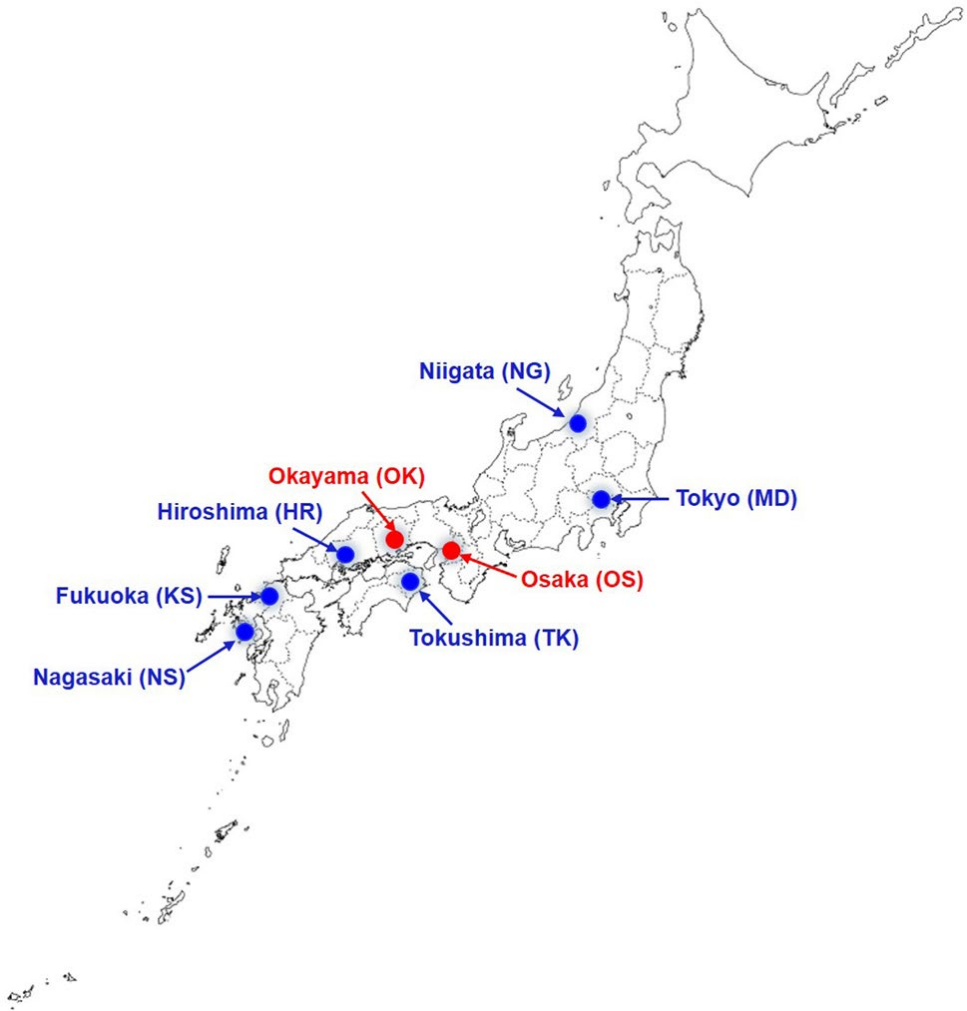
Geographic locations of the eight study sites in Japan where subjects were recruited: Hiroshima University Hospital, Hiroshima (HR); Kyushu University Hospital, Fukuoka (KS); Nagasaki University Hospital, Nagasaki (NS); Niigata University Medical and Dental Hospital, Niigata (NG); Okayama University Hospital, Okayama (OK); The University of Osaka Dental Hospital, Osaka (OS); Tokushima University Hospital, Tokushima (TK); and Institute of Science Tokyo Hospital, Tokyo (MD). The two-letter codes in parentheses represent the strain codes used for *S. mutans* isolates collected from each site. Locations highlighted in red (Okayama and Osaka) indicate the cities where caries indices were recorded and analyzed in Fig. 2

A total of 1248 subjects, 1–90 years of age, enrolled in this study. These subjects lived in eight cities in Japan: Hiroshima, Fukuoka, Nagasaki, Niigata, Okayama, Osaka, Tokushima, and Tokyo (Fig. 1). Research sites for subject recruitment from these cities were Hiroshima University Hospital (HR), Kyushu University Hospital (KS), Nagasaki University Hospital (NS), Niigata University Medical and Dental Hospital (NG), Okayama University Hospital (OK), The University of Osaka Dental Hospital (OK), Tokushima University Hospital (TK), and the Institute of Science Tokyo Hospital (MD), respectively. The research protocol for the present study was approved by the Ethics Committee of the Graduate School of Dentistry, The University of Osaka (Ethic No. R1-E51-1), and prior written informed consent was obtained from participants and/or the legal guardians of participants.

For all subjects, data regarding age, sex, and the number of teeth were collected. For the subjects from Okayama and Osaka, caries experience data were also recorded by the dmft and DMFT indices established by the World Health Organization for decayed, missing, and filled teeth in primary and permanent teeth, respectively. In addition, the numbers of decayed primary and permanent teeth were reported as dt and DT.

The exclusion criteria were subjects who had received antibiotics and those with incomplete data regarding the subject’s general or oral information, as indicated above.

### Specimen collection and isolation of *S. mutans*

For each subject, a sterile swab was used to collect the whole dental plaque, which was kept in Amies transport medium (Eiken Chemical Co. Ltd., Tochigi, Japan) and sent to the laboratory. The collected dental plaque was cultured on Mitis Salivarius agar (Difco Laboratories, Detroit, MI, USA) containing bacitracin (0.2 U/mL; Sigma Chemical Co., St. Louis, MO, USA), 0.001% (v/v) tellurite solution (Becton, Dickinson and Co., Sparks, MD, USA), and 15% (w/v) sucrose (MSB agar plate) under anaerobic conditions at 37 °C for 2 days.

Five colonies growing on the MSB agar plate were randomly picked based on colony morphology and kept as stocks in brain-heart infusion (BHI) broth (Difco Laboratories) with 50% glycerol. The genomic DNA of these stocks was then extracted using the protocol for gram-positive bacteria [7]. The obtained DNA was confirmed to be *S. mutans* by PCR using species-specific primers (Table S1) [29], with *S. mutans* strain MT8148 [30] serving as a positive control.

### Determination of *S. mutans* harboring genes encoding collagen-binding proteins

The genomic DNA of all *S. mutans* isolates was tested for the presence of *cnm* and *cbm* genes encoding collagen-binding proteins by PCR using specific primers, as previously described [11, 31]. Briefly, for the *cnm* gene, the PCR primers employed were *cnm*−1 F (5′- GAC AAA GAA ATG AAA GAT GT-3′) and *cnm*−1R (5′- GCA AAG ACT CTT GTC CCT GC-3′). The reaction mixture (20 µL) contained 0.5 U Ex Taq™ DNA polymerase (Takara Bio Inc., Shiga, Japan), 2 µL of 10× Ex Taq buffer (Takara Bio) containing 20 mM Mg^2+^, 1.6 µL dNTPs (2.5 mM), 0.5 µL each of forward and reverse primers (20 µM), 40 ng DNA, and 13.3 µL sterilized water. For the *cbm* gene, the PCR primers *cbm*−1 F (5′- GAC AAA CTA ATG AAA TCT AA-3′) and *cbm*−3R (5′-GCA AAA ACT GTT GTC CCT GC-3′) were utilized using the same reaction mixture as described for the *cnm* gene. Thermocycling for both genes was carried out as follows: 95 °C for 4 min; followed by 30 cycles at 95 °C for 30 s, 53 °C for 30 s, and 72 °C for 2 min; with a final extension step at 72 °C for 7 min. The PCR products were separated by agarose gel electrophoresis and visualized by staining with ethidium bromide.

*S. mutans* strain TW295 [32] possessing the *cnm* gene and *S. mutans* strain SA31 [28] possessing the *cbm* gene were used as positive controls, while a *S. mutans* strain lacking both of these genes (MT8148) [30] was used as a negative control.

### MLST analysis of *S. mutans*

*S. mutans* isolates were genotyped using the MLST method, based on the Nakano scheme with some modifications [28, 33]. From each city, we randomly selected five *cnm*-positive *S. mutans* isolates and five *cnm*-negative *S. mutans* isolates. These were obtained from five subjects harboring *cnm*-positive *S. mutans* and five subjects lacking *cnm*-positive *S. mutans*, respectively.

Genomic DNA of all isolates were amplified for the internal fragments of eight housekeeping genes: transketolase (*tkt*), glutamine synthetase type I (*glnA*), glutamate synthetase (*gltA*), glucose kinase (*glk*), shikimate 5-dehydrogenase (*aroE*), glutamate racemase (*murI*), signal peptidase I (*lepC*), and DNA gyrase A subunit (*gyrA*). After checking PCR products by agarose gel electrophoresis and staining with ethidium bromide, the products were purified with ExoSAP-IT (USB Corp., Cleveland, OH, USA), and sequenced using their respective PCR primers. The results obtained were registered in the GenBank database (accession numbers: PV492251–PV492329 for *tkt*; PV492669–PV492747 for *glnA*; PV492748–PV492826 for *gltA*; PV492827–PV492905 for *glk*; PV492906–PV492984 for *aroE*; PV492985–PV493063 for *murI*; PV524416–PV524494 for *lepC*; and PV524495–PV524573 for *gyrA*).

Each unique nucleotide sequence identified in a housekeeping gene was assigned a specific allele number. For each *S. mutans* isolate, the combination of allele numbers of the eight genes defined the allelic profile and the sequence type (ST). To maintain consistency with existing data, nucleotide sequences of the housekeeping gene fragments analyzed in this study were compared against the Oral *Streptococcus* PubMLST database (http://pubmlst.org/oralstrep/) [34]. Identical sequences were assigned the corresponding allele numbers and STs, while novel sequences were submitted to the database for designation.

The allelic profiles of the analyzed *S. mutans* isolates were further examined using the START (sequence type analysis and recombinational tests) program [35], generating a matrix of pairwise differences in the allelic profiles. A dendrogram was then constructed from this matrix using the unweighted pair group method with arithmetic mean (UPGMA). To identify related STs, the goeBURST (global optimal enhanced based upon related sequence types) algorithm, implemented in the PHYLOViZ program [36], was applied. Isolates sharing identical alleles at six or more loci were classified as members of a “clonal complex” (CC) or a closely-related group of STs that have evolved from a common ancestor.

### Serotype determination

*S. mutans* strains that were grouped into CCs by MLST analysis, as well as those sharing the same ST but differing in the presence of the *cnm* gene, were further analyzed for serotype by PCR using serotype-specific primer sets (Table S1), as previously described [37, 38]. Reference *S. mutans* strains MT8148, NN2002, LJ24, and TW295, corresponding to serotypes *c*, *e*, *f*, and *k*, respectively [28, 32], were used as positive controls in each PCR assay.

#### Statistical analysis

Statistical analyses were conducted using SPSS software version 18.0 (SPSS Corp., Chicago, IL, USA) and GraphPad Prism version 5.0 (GraphPad Software Inc., La Jolla, CA, USA). The categorical variables are presented as numbers and percentages, and the continuous variables are presented as means with standard deviations and medians. Associations between categorical variables were evaluated using the χ² test. Continuous variables were compared using the Mann–Whitney U test (Mann-U) or the Kruskal–Wallis test, as appropriate. A *p-*value of less than 0.05 was considered statistically significant.

## Results

### Detection of *S. mutans* and the genes encoding collagen-binding proteins

Among the 1248 subjects enrolled (Table 1), *S. mutans* was detected in 523 (41.9%). The minimum age of bacterial detection was 3 years and the maximum age was 90 years. The median age of the *S. mutans* detection group was significantly higher than that of the group lacking *S. mutans* detection (36 vs. 26; *p* = 0.001, Mann-U test). However, no significant difference was found in the total number of teeth regarding *S. mutans* detection (*p* = 0.081, Mann-U test). The age of subjects from Okayama and Osaka was lower than that of those from other cities (*p* < 0.001, Kruskal–Wallis test) (Table 1). There was no significant difference among the cities regarding the sex of participants (*p* = 1.000, χ² test).

**Table 1:** Japanese subjects from eight cities included in this study

City (Strain code)	Number of subjects	Sex F : M	Age Mean ± SD (Median)	Total number of teeth Mean ± SD (Median)	*S. mutans *detection (%)	*cnm *detection (%)	*cbm *detection (%)
Hiroshima (HR)	118	59 : 59	50.8 ± 17.9 (49.5)	25.9 ± 4.9 (28.0)	71/118 (60.2)	6/118 (5.1)	1/118 (0.8)
Fukuoka (KS)	190	97 : 93	50.2 ± 17.4 (51.0)	25.3 ± 4.7 (27.0)	87/190 (45.8)	9/190 (4.7)	2/190 (1.1)
Nagasaki (NS)	94	49 : 45	51.7 ± 17.5 (56.0)	24.3 ± 5.3 (26.0)	39/94 (41.5)	6/94 (6.4)	2/94 (2.1)
Niigata (NG)	123	62 : 61	50.6 ± 18.2 (50.0)	26.1 ± 4.1 (28.0)	57/123 (46.3)	10/123 (8.1)	1/123 (0.8)
Okayama (OK)	250	123 : 127	9.5 ± 3.7 (9.0)	23.9 ± 3.1 (24.0)	73/250 (29.2)	4/250 (1.6)	2/250 (0.8)
Osaka (OS)	240	121 : 119	9.6 ± 3.7 (9.5)	23.6 ± 3.0 (24.0)	102/240 (42.5)	12/240 (5.0)	0/240 (0)
Tokushima (TK)	115	58 : 57	50.2 ± 17.4 (51.0)	24.5 ± 5.5 (27.0)	56/115 (48.7)	14/115 (12.2)	0/115 (0)
Tokyo (MD)	118	58 : 60	50.1 ± 17.5 (49.5)	24.2 ± 6.5 (27.0)	38/118 (32.2)	7/118 (5.9)	0/118 (0)
**Total**	**1248**	**627 : 621**	**34.4 ± 24.4** **(30.0)**	**24.6 ± 4.5** **(26.0)**	**523/1248** **(41.9)**	**68/1248** **(5.4)**	**8/1248** **(0.6)**

For the detection of *S. mutans* harboring genes encoding collagen-binding proteins, *cnm*-positive *S. mutans* was found in 68 subjects (68/1248; 5.4%), whereas only eight subjects possessed *cbm*-positive *S. mutans* (8/1248; 0.6%) (Table 1). No single *S. mutans* isolate carrying both *cnm* and *cbm* genes was detected, nor was any subject found to possess both *cnm*-positive and *cbm*-positive *S. mutans*. Among the 68 subjects with *cnm*-positive *S. mutans*, all isolates from 42 subjects were *cnm*-positive strains. In contrast, mixed populations containing both *cnm*-positive and *cnm*-negative *S. mutans* were identified in the remaining 26 subjects. Regarding the eight subjects from whom *cbm*-positive *S. mutans* were detected, five exclusively carried *cbm*-positive strains among all *S. mutans* collected. *S. mutans* harboring the *cnm* gene was present in all cities, whereas subjects with *cbm*-positive *S. mutans* were not detected in Osaka, Tokushima, or Tokyo. Because the *cnm* gene was more frequently detected than the *cbm* gene in this study population, we further evaluated the information on individuals harboring *S. mutans* with the *cnm* gene. The youngest participant possessing *S. mutans* with the *cnm* gene was a 3-year-old girl from Osaka, and the oldest participants possessing *S. mutans* with the *cnm* gene were two 85-year-old men from Niigata.

When considering only the 523 subjects with *S. mutans* (Table 2), the presence of the *cnm* gene was 13.0%. There was no difference in the total number of teeth between the group with *cnm*-positive *S. mutans* and the group with *S. mutans* without the *cnm* gene (*p* = 0.088, Mann-U test). However, the ages of subjects with *cnm*-positive *S. mutans* were higher than those of subjects having only *S. mutans* without the *cnm* gene (53 vs. 35; *p* = 0.002, Mann-U test). The *cnm* detection rates were lowest in Okayama and highest in Tokushima, either when all 1248 subjects (Table 1) or only the 523 subjects harboring *S. mutans* (Table 2) were considered.

**Table 2:** Ages of 523 subjects harboring *S. mutans* based on the presence of the *cnm* gene

City (Strain code)	Number of subjects having *S. mutans*	Total number of teeth Mean ± SD (Median)	*cnm *detection (%)	Age of subjects with the *cnm*-positive *S. mutans *Mean ± SD (Median)	Age of subjects without the *cnm*-positive *S. mutans *Mean ± SD (Median)
Hiroshima (HR)	71	25.9 ± 5.3 (28.0)	6/71 (8.5)	49.3 ± 22.3 (52.5)	51.2 ± 18.5 (49.0)
Fukuoka (KS)	87	25.0 ± 5.1 (27.0)	9/87 (10.3)	53.9 ± 12.7 (60.0)	49.7 ± 17.8 (47.5)
Nagasaki (NS)	39	24.12 ± 5.1 (26.0)	6/39 (15.4)	58.0 ± 8.4 (57.0)	49.6 ± 17.2 (49.0)
Niigata (NG)	57	25.5 ± 4.9 (28.0)	10/57 (17.5)	66.5 ± 15.8 (69.0)	50.5 ± 15.2 (50.0)
Okayama (OK)	73	24.3 ± 2.9 (24.0)	4/73 (5.5)	8.3 ± 4.6 (6.5)	9.9 ± 3.7 (10.0)
Osaka (OS)	102	23.9 ± 2.9 (24.0)	12/102 (11.8)	8.5 ± 3.2 (9.5)	9.7 ± 3.5 (10.0)
Tokushima (TK)	56	24.1 ± 5.5 (26.0)	14/56 (25.0)	58.6 ± 11.4 (60.0)	49.1 ± 17.8 (48.0)
Tokyo (MD)	38	25.6 ± 6.4 (28.0)	7/38 (18.4)	54.3 ± 17.4 (46.0)	47.2 ± 17.3 (45.0)
**Total**	**523**	**24.7 ± 4.7** **(26.0)**	**68/523** **(13.0)**	**46.0 ± 24.8** **(53.0)**	**35.8 ± 23.8** **(35.0)**

### Decayed teeth, caries experience and *S. mutans* with the *cnm* gene

**Fig. 2:**
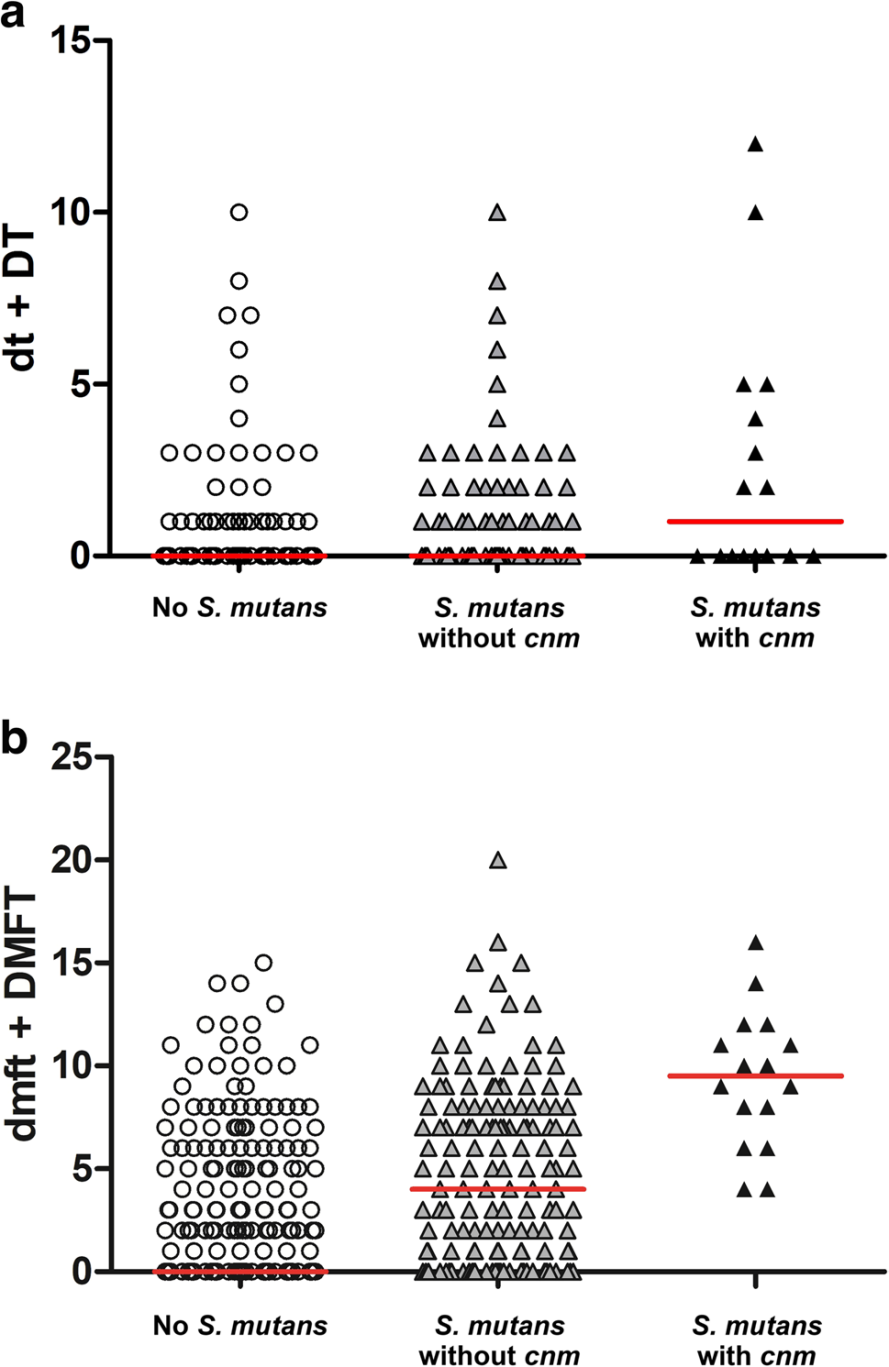
Caries indices among 490 subjects from Okayama (*n* = 250) and Osaka (*n* = 240): (**a**) total number of decayed teeth (dt + DT), and (**b**) total number of decayed, missing, and filled teeth (dmft + DMFT) in primary and permanent dentition. Red horizontal lines indicate median values. Subjects without *S. mutans* (*n* = 315) exhibited significantly lower scores than those with *S. mutans* lacking the *cnm* gene (*n* = 159), whereas the highest dt + DT and dmft + DMFT scores were observed in subjects harboring *cnm*-positive *S. mutans* (*n* = 16) (*p* < 0.001, Kruskal-Wallis test)

Data on the number of decayed, missing, and filled teeth were analyzed for 490 subjects from Okayama and Osaka in relation to the presence of *S. mutans* and the *cnm* gene. Fig. 2 shows that subjects without *S. mutans* had the lowest number of decayed teeth and the lowest dmft + DMFT scores (*p* < 0.001, Kruskal–Wallis test). A comparison of both caries prevalence and caries experience also revealed that subjects with *S. mutans* possessing the *cnm* gene exhibited higher numbers of carious teeth and higher dmft + DMFT scores than those with *S. mutans* lacking the *cnm* gene.

When comparing Okayama and Osaka, the subjects from Okayama had a lower *S. mutans* detection rate (29.2% vs. 42.5%; *p* = 0.002, χ² test) and lower dmft + DMFT scores (1 vs. 2; *p* = 0.003, Mann-U test) than those in Osaka. The *cnm* detection rates in all subjects between these two cities were different (Okayama 1.6% vs. Osaka 5.0%; *p* = 0.034, χ² test), although there was no statistical difference when comparing the *cnm* detection rates from only subjects possessing *S. mutans* (Okayama 5.5% vs. Osaka 11.8%; *p* = 0.155, χ² test).

### Genotyping and clonal relationship of *S. mutans* with and without the *cnm* gene by MLST

We aimed to analyze five *cnm*-positive and five *cnm*-negative *S. mutans* isolates from eight cities. However, only four subjects from Okayama were found to harbor *S. mutans* with the *cnm* gene (Table 2). Consequently, 39 *cnm*-positive and 40 *cnm*-negative *S. mutans* isolates, derived from 39 to 40 subjects with and without the *cnm* gene, respectively, were included in the MLST analysis.

These 79 *S. mutans* isolates were classified into 53 unique STs, 41 of which (ST353–ST393) were novel and had not been previously recorded in the Oral Streptococcus PubMLST database (Table S2). Among these, ST355 was the most common ST identified in this population. A total of 17 STs included more than one *S. mutans* strain. Notably, most strains sharing the same ST also shared the same presence of the *cnm* gene (14/17 STs). The exceptions were three STs—ST276, ST277, and ST355—that contained both *cnm*-positive and *cnm*-negative *S. mutans* strains. There was no difference in the number of STs identified between *cnm*-positive and *cnm*-negative *S. mutans*. A total of 28 STs were identified from the 39 *cnm*-positive strains, and 28 STs were also identified from the 40 *cnm*-negative strains.

**Fig. 3:**
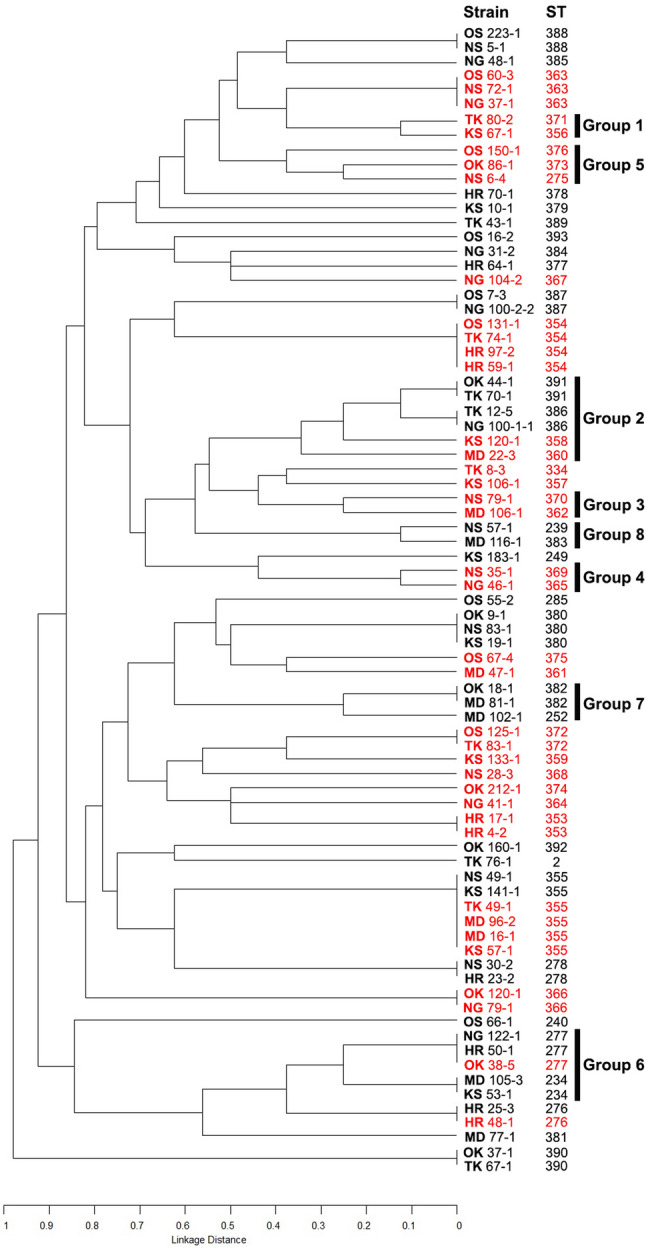
Dendrogram and clonal complex (CC) grouping of all sequence types (STs) based on MLST analysis of 79 *S. mutans* strains. These strains were isolated from 39 subjects harboring *cnm*-positive *S. mutans* and 40 subjects lacking *cnm*-positive strains. Clonal relationships were inferred from the allelic profiles of eight housekeeping genes. Red color indicates the *cnm*-positive strains. The two-letter codes at the beginning of each strain indicate the eight study sites in Japan where strains were collected: Hiroshima University Hospital, Hiroshima (HR); Kyushu University Hospital, Fukuoka (KS); Nagasaki University Hospital, Nagasaki (NS); Niigata University Medical and Dental Hospital, Niigata (NG); Okayama University Hospital, Okayama (OK); The University of Osaka Dental Hospital, Osaka (OS); Tokushima University Hospital, Tokushima (TK); and Institute of Science Tokyo Hospital, Tokyo (MD)

The clonal relationships of all 53 STs identified are shown in Fig. 3. There was no CC related to *S. mutans* from each city; however, MLST analysis showed the clonal relationships of the *cnm*-positive strains from various cities of Japan (Groups 1, 3, 4, and 5). In addition, Groups 7 and 8 contained the *cnm*-negative *S. mutans* from more than one city. As for the remaining two CCs (Groups 2 and 6), these CCs contained both *cnm*-positive and *cnm*-negative *S. mutans* strains, and the sources of such strains were also mixed.

The serotypes of *S. mutans* strains in each CC were different (Table 3). The strains in the *cnm*-positive CCs (Groups 1, 3, 4, and 5) were almost all serotypes *f* and *k*, whereas the *cnm*-negative CCs (Groups 7 and 8) contained only serotype *c*. For the CCs with both *cnm*-positive and *cnm*-negative *S. mutans*, Groups 2 and 6 contained the strains in serotypes *c*, *e*, and *f*. The serotype information of three STs—ST276, ST277, and ST355—showing both *cnm*-positive and *cnm*-negative *S. mutans*, is presented in Table 4. Both *cnm*-positive and *cnm*-negative *S. mutans* classified as ST276 were serotype *c*, while all *cnm*-positive and *cnm*-negative strains of ST355 were serotype *e*. Nevertheless, ST277 contained both serotypes *e* and *f*.

**Table 3:**
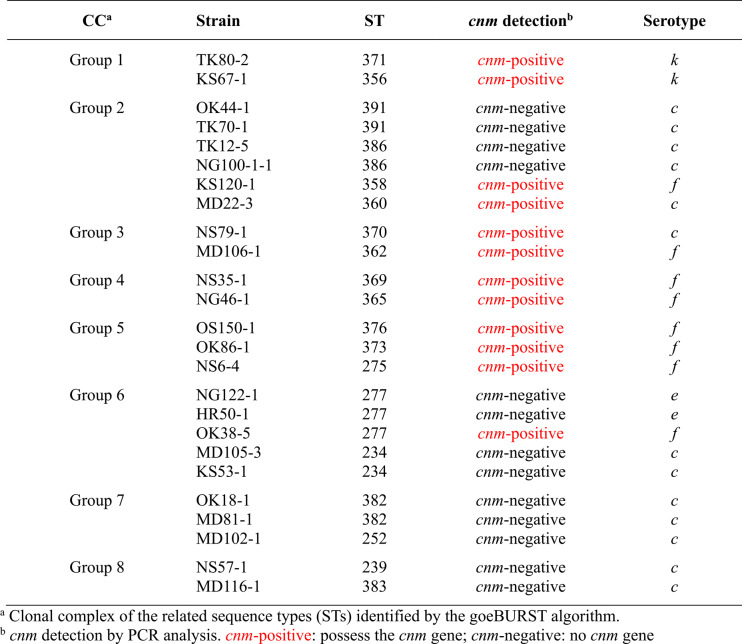
Serotypes of *S. mutans* in each clonal complex based on MLST analysis

**Table 4:**
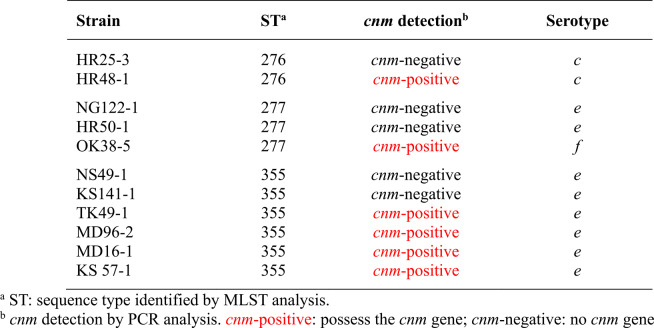
Serotypes of *S. mutans* in three sequence types with both *cnm*-positive and *cnm*-negative strains

## Discussion

In this study population (*n* = 1248), the subjects harboring *S. mutans* were significantly older than those without this bacterium. This finding supports the notion that the colonization of *S. mutans* tends to accumulate with age, as previously reported [39–42]. Although classic studies associated *S. mutans* colonization with tooth eruption [39, 43, 44], its presence in predentate infants indicated that *S. mutans* could colonize non-tooth surfaces, such as the oral mucosa and tongue, under certain conditions [41, 42, 45, 46]. *S. mutans* exhibits efficient colonization strategies, including both sucrose-independent and sucrose-dependent mechanisms that facilitate adherence to tooth surfaces [2, 47, 48]. Nevertheless, our study found no significant difference in the number of teeth present in individuals with or without *S. mutans* colonization. This suggests that the presence of *S. mutans* is not solely dependent on the number of tooth surfaces but may also involve other factors, such as oral hygiene, dietary habits, or environmental microbial interactions [45, 49–51].

Among the 523 subjects harboring *S. mutans*, a significant difference in age was observed when considering the presence of the *cnm* gene. Subjects with *cnm*-positive *S. mutans* were older than those with *cnm*-negative strains. The *cnm*-positive *S. mutans* strains have been frequently associated with systemic diseases, such as infective endocarditis, IgA nephropathy, non-alcoholic steatohepatitis, cerebral hemorrhage, and cerebral microbleeds [18, 20, 52–55]. This finding may suggest that older individuals with *cnm*-positive *S. mutans* are at higher risk of developing systemic complications associated with this bacterium. Alternatively, the higher ages observed in the *cnm*-positive group might reflect a greater prevalence of underlying conditions that were not documented in this study, as no medical history was collected from the participants. Further investigation, including comprehensive medical histories and longitudinal studies, is necessary to clarify whether the presence of *cnm*-positive *S. mutans* in older individuals directly increases their risk of systemic disease or is simply a marker of existing health conditions. Research addressing this issue is currently underway by our research team.

More than 10 and 20 years have passed since the *cnm* and *cbm* genes were first described in *S. mutans* [9, 11], and since then, multiple studies have reported the presence of *S. mutans* strains harboring these genes. Such research studies spanned diverse specimen types and geographic regions, leading to variations in detection rates [11, 13, 15, 56–60], although both *cnm* and *cbm* genes were consistently identified within a minor population of *S. mutans* [7, 11, 13, 57–63]. In *S. mutans* from saliva and dental plaque, most studies reported a lower prevalence of the *cbm* gene than of the *cnm* gene [11, 57, 59, 63, 64]. However, a higher prevalence of *cbm* than of *cnm* has been noted in some studies evaluating the prevalence of *cnm* and *cbm* in heart valves from infective endocarditis patients [56], endodontic infections [14], and dentin carious lesions [60], highlighting variations and potential niche-specific adaptations. In the present study using dental plaque as the specimen, *cnm*-positive *S. mutans* was detected in all eight cities of Japan, with prevalence rates ranging from 1.6% to 12.2% of the total study population and from 5.5% to 25% among subjects harboring *S. mutans*. By contrast, *cbm*-positive *S. mutans* was found at lower rates and was entirely absent in some cities, such as Osaka, Tokushima, and Tokyo. Previous research suggests evolutionary differences between *S. mutans* strains carrying these *cnm* and *cbm* genes [8], while only the *cbm* gene is genetically linked to a putative type VII secretion system found in Mycobacteria and a few other Gram-positive bacteria [12], thus emphasizing the need for additional study to compare these two collagen-binding genes.

We further analyzed *cnm*-positive *S. mutans* based on its higher detection frequency compared with the *cbm* gene in this study population. Its distribution along with the oral information obtained from participants confirmed the lack of correlation between total tooth number and the detection of *S. mutans* with the *cnm* gene. However, a correlation was detected between subjects harboring *S. mutans* with the *cnm* gene and higher numbers of carious teeth and greater caries experience scores than those with only *S. mutans* lacking the *cnm* gene, while subjects lacking *S. mutans* exhibited the lowest values for both measures. This analysis was based on the dmft and DMFT indices of 490 subjects aged 1–19 years from Okayama and Osaka. We were unable to include caries experience data from participants in the other cities, where the mean age was approximately 50, because we were not able to confirm the specific causes of tooth extractions and fillings for these subjects. As a result, it was not possible to reliably distinguish tooth loss or fillings caused by dental caries from those resulting from other conditions, such as periodontitis, orthodontic treatment, or non-carious reasons like tooth attrition or abrasion. Based on the ambiguity of these data, we decided to exclude the caries experience data from these cities from the analysis.

Our finding of an association between high caries prevalence and caries experience with *S. mutans* harboring the *cnm* gene in Japanese children and adolescents in the present study was consistent with previous studies in Canada, Iran, Japan, and Sweden [52, 57, 61, 62]. In some studies, the detection of *cnm*-positive *S. mutans* was also linked to increased caries risk [57] and associated with caries recurrence [64]. Moreover, Cnm protein encoded by the *cnm* gene was proposed as a colonization factor contributing to the pathogenicity of a subset of *S. mutans* strains in the oral cavity [65]. Thus, our findings support the significant role of *S. mutans*, particularly *cnm*-positive *S. mutans*, in dental caries. A comparison between children from Okayama and Osaka revealed a consistent trend; subjects from Okayama exhibited both lower caries experience and a lower prevalence of *cnm*-positive strains than those from Osaka. The association between high caries levels and *cnm*-positive *S. mutans* was further demonstrated by the youngest subject harboring this strain—a 3-year-old girl from Osaka who presented with carious lesions in 12 of her deciduous teeth (data not shown).

Although differences in the detection rates of the *cnm* gene were observed across cities, MLST analysis revealed no city-specific CC among the *cnm*-positive *S. mutans* strains. This finding suggests that *cnm*-positive strains from the eight Japanese cities shared evolutionary relationships based on their core genomes, as determined by the analysis of eight housekeeping genes. Consistent with our results, previous studies of *S. mutans* from various countries have also reported shared MLST allelic profiles and STs among isolates from individuals in different geographic regions [33, 66]. In the present study, four *cnm*-positive strains were identified as ST275, ST276, ST277, and ST334—STs that have also been reported and deposited in the Oral *Streptococcus* PubMLST database (http://pubmlst.org/oralstrep/) from isolates originating in Brazil, Iceland, Sweden, the United Kingdom, and the United States [34]. Thus, *S. mutans* strains with the same MLST profiles as *cnm*-positive strains found in Japan have also been identified in other parts of the world.

When all eight CCs identified by MLST were analyzed for their *cnm* gene status, four CCs (Groups 1, 3, 4, and 5) consisted exclusively of *cnm*-positive *S. mutans* strains, while two CCs (Groups 7 and 8) were composed solely of *cnm*-negative strains. These findings suggest that clonal relationships based on core housekeeping genes can partially reflect the distribution of the *cnm* gene within *S. mutans* populations. This observation is consistent with previous research that indicated the potential for identifying *cnm*-positive clones using MLST [7]. However, our results also revealed two CCs (Groups 2 and 6) containing both *cnm*-positive and *cnm*-negative *S. mutans*. Furthermore, three STs identified in this study (ST276, ST277, and ST355) were designated for either *cnm*-positive or *cnm*-negative strains. This inconsistency between *cnm* gene presence and MLST profiles, including defined STs and clonal relationships, can be attributed to the non-core nature of the *cnm* gene itself. The *cnm* gene is located upstream of the *pgfS* gene (SMU.2067), which encodes a putative glycosyltransferase and is part of the *S. mutans* core genome [12]. The product of this core *pgfS* gene is responsible for the glycosylation of the Cnm protein, a process crucial for the binding of Cnm to collagen and the invasion of host cells [67]. The observed discrepancies in *cnm* distribution despite shared core genomic profiles suggest that horizontal gene transfer (HGT) plays a significant role in the dissemination of this non-core gene. Such HGT events provide *S. mutans* with niche-specific benefits, enhancing adaptability and virulence in diverse environments, while contributing to genomic variations within clonally-related populations.

The observed serotype distribution within the CCs and STs provides insights into the complex relationship between core genome evolution, the non-core *cnm* gene, and serotypes in *S. mutans*. Our analysis revealed a striking association: *cnm*-positive CCs (Groups 1, 3, 4, and 5) predominantly comprised serotypes *f* and *k*, whereas *cnm*-negative CCs (Groups 7 and 8) exclusively comprised serotype *c* strains. While this observation suggests a degree of correlation between serotype, core genomic lineage, and *cnm* presence, it is important to note that exceptions exist. For example, in the *cnm*-positive CC, a serotype *c* strain (ST370, Group 3) was also identified. The consistent presence of *cnm* in these four CCs, coupled with their specific serotypes, may indicate that these lineages have undergone successful clonal expansions where this genetic combination has become established. Notably, these findings align with previous reports indicating a higher prevalence of the *cnm* gene among the minor serotypes of *S. mutans*, specifically serotypes *f* and *k* [7, 58, 62]. However, this association is not absolute. The CCs exhibiting a mixed *cnm* status (Groups 2 and 6) were heterogeneous in their serotype composition, including strains of serotypes *c*, *e*, and *f*. Moreover, the detailed serotype information for three STs with mixed *cnm* status in Table 4. also showed that ST276, despite containing both *cnm*-positive and *cnm*-negative *S. mutans*, was exclusively serotype *c*. Similarly, all *cnm*-positive and *cnm*-negative strains within ST355 belonged solely to serotype *e*. Only ST277 displayed a mix of serotypes (*e* and *f*) alongside its mixed *cnm* status. Therefore, the core genome, as tracked by MLST, does not seem to correlate with serotype.

Serotypes of *S. mutans*, specifically *c*, *e*, *f*, and *k*, are defined by the chemical composition of rhamnose–glucose polymers on the bacterial surface, with their biosynthesis involving several genes within specific pathways [24]. Although serotype *c* is recognized as the major *S. mutans* population, both this study and previous reports consistently showed higher rates of *cnm* detection in the minor serotypes [3, 7, 8, 62]. This prevalence pattern has led to the hypothesis that certain *S. mutans* serotypes might possess a greater pathogenic potential for systemic disease [16, 24, 56, 68]. Although our study observed the clonal expansion of serotypes *f* and *k* of *S. mutans* possessing the *cnm* gene, the findings also suggest that *cnm* acquisition via HGT acts as a primary driver of its non-uniform distribution in some clones, irrespective of their serotype. This highlights the dynamic nature of the *S. mutans* accessory genome, where non-core genes such as the *cnm* gene can confer specific advantages, thereby transcending traditional clonal and serological classifications. Further study regarding the stability of the *cnm* gene in the *S. mutans* genome, as well as the persistence of *S. mutans* strains possessing this gene in various areas of the oral cavity, is of interest and may provide crucial insights into the long-term ecological fitness and pathogenic potential of *cnm*-positive *S. mutans*.

## Conclusions

This study elucidated the distribution patterns of *S. mutans* strains harboring collagen-binding adhesin genes across multiple cities in Japan. We observed that *cnm*-positive *S. mutans* was present in all evaluated cities, whereas *cbm*-positive *S. mutans* was not detected in some regions. Correlating these findings with demographic and oral examination data, we found that subjects with *cnm*-positive *S. mutans* were significantly older than those carrying only *cnm*-negative strains. Furthermore, while no association was found with the number of teeth, the presence of *cnm*-positive *S. mutans* was significantly linked to higher caries prevalence and caries experience. MLST analysis, based on core housekeeping genes, revealed the evolutionary relationships among *cnm*-positive *S. mutans* strains across the eight Japanese cities, notably without clear evidence of region-specific clustering. Our findings confirm that clonal relationships, as defined by core genes, can partially reflect the distribution of *cnm* within *S. mutans* populations, particularly for those exhibiting exclusive *cnm*-positive or *cnm*-negative status. However, the observed inconsistencies between serotypes, *cnm* presence, and MLST-defined clones further underscore the non-core nature of the *cnm* gene.

## Supplementary Information

Supplementary Material 1

Supplementary Material 2

## Data Availability

All data generated or analyzed during this study are included in the article and its supplementary files. All nucleotide sequences obtained during MLST analysis have been registered in the GenBank database (accession numbers PV492251–PV492329 for **tkt**; PV492669–PV492747 for **glnA**; PV492748–PV492826 for **gltA**; PV492827–PV492905 for **glk**; PV492906–PV492984 for **aroE**; PV492985–PV493063 for **murI**; PV524416–PV524494 for **lepC**; and PV524495–PV524573 for **gyrA**). Further enquiries can be directed to the corresponding author.

## Abbreviations

CC: Clonal complex
HGT: Horizontal gene transfer
MLST: Multilocus sequence typing
ST: Sequence type
