# Supplementary material for: Evaluation of Streptococcus mutans strains possessing genes encoding collagen-binding proteins in the Japanese population
Source: BMC Oral Health. 2025 Nov 25;25:1908. doi: 10.1186/s12903-025-07276-5 (PMC12703920; doi:10.1186/s12903-025-07276-5)
Supplement: Supplementary file 1 — Supplementary Material 1 [file 12903_2025_7276_MOESM1_ESM.docx]

**Table S1** Primer sets used in the present study

| Purpose | Name | Sequence (5′ to 3′) | References |
| --- | --- | --- | --- |
| Detection of *S. mutans* | MKD-F | GGC ACC ACA ACA TTG GGA AGC TCA GTT | [29] |
|  | MKD-R | GGA ATG GCC GCT AAG TCA ACA GGA T |  |
| Detection of *cnm* gene | *cnm*-1F | GAC AAA GAA ATG AAA GAT GT | [31] |
|  | *cnm*-1R | GCA AAG ACT CTT GTC CCT GC |  |
| Detection of *cbm* gene | *cbm*-1F | GAC AAA CTA ATG AAA TCT AA | [11] |
|  | *cbm*-3R | GCA AAA ACT GTT GTC CCT GC |  |
| Multilocus sequence typing of *S. mutans* | *tkt*/F | CAG ATT TAT CGG TTA ATG CCA TTC G | [33] |
|  | *tkt*/R | TTA GTT GGA GCA CCG TAG CC |  |
|  | *glnA*/F | ACA AAG CGA TGT TTG ATG GCT |  |
|  | *glnA*/R | GCG TTC TTA CCA TCA CTG CC |  |
|  | *gltA*/F | TTG AGA CAG ATG CCT GTG GG |  |
|  | *gltA*/R | AAG CAT GCA GCA TTC CCT TA |  |
|  | *glk*/F | AGG GAT TGA TCT TGG TGG AAC A |  |
|  | *glk*/R | AAA TGA CGT GCA ACA CGG AC |  |
|  | *aroE*/F | ATG CCT TAC AAG CAG GCA GT |  |
|  | *aroE*/R | AGC CTG CCA GAT TTC CTG AC |  |
|  | *murI*/F | GAC CTA TTG GTT TTT TAG ACT CCG |  |
|  | *murI*/R | TCA ATT TTC CCC ACC AGA GGA |  |
|  | *lepC*/F | AGA ATG GGG CCT TTT CTT GGT C |  |
|  | *lepC*/R | GCC AAA AGC GGA ATT TAA CTT CAC C |  |
|  | *gyrA*/F | TCG GGC TCT TCC AGA TGT TC |  |
|  | *gyrA*/R | AGG CGC GAT GTA TAC CCG AT |  |
| Determination of *S. mutans* serotype | SC-F | CGG AGT GCT TTT TAC AAG TGC TGG | [37] |
|  | SC-R | AAC CAC GGC CAG CAA ACC CTT TAT |  |
|  | SE-F | CCT GCT TTT CAA GTA CCT TTC GCC |  |
|  | SE-R | CTG CTT GCC AAG CCC TAC TAG AAA |  |
|  | SF-F | CCC ACA ATT GGC TTC AAG AGG AGA |  |
|  | SF-R | TGC GAA ACC ATA AGC ATA GCG AGG |  |
|  | CEFK-F | ATT CCC GCC GTT GGA CCA TTC C | [38] |
|  | CEF-R | CCG ACA AAG ACCATT CCA TCT C |  |
|  | K-R | CCA ATG TGA TTC ATC CCA TCA C |  |
