# Supplementary material for: Evaluation of Streptococcus mutans strains possessing genes encoding collagen-binding proteins in the Japanese population
Source: BMC Oral Health. 2025 Nov 25;25:1908. doi: 10.1186/s12903-025-07276-5 (PMC12703920; doi:10.1186/s12903-025-07276-5)
Supplement: Supplementary file 2 — Supplementary Material 2 [file 12903_2025_7276_MOESM2_ESM.docx]

**Table S2** Allelic profiles and sequence types (STs) of 79 *S. mutans* strains isolated from 79 Japanese subjects in this study

| ST | Allelic profile^a^ | | | | | | | | No. of strains detected  (*cnm*-positive/*cnm*-negative) |
| --- | --- | --- | --- | --- | --- | --- | --- | --- | --- |
|  | *tkt* | *glnA* | *gltA* | *glk* | *aroE* | *murI* | *lepC* | *gyrA* |  |
| 2 | 1 | 1 | 1 | 13 | 1 | 1 | 1 | 1 | 1 (0/1) |
| 234 | 6 | 4 | 42 | 4 | 5 | 5 | 1 | 4 | 2 (0/2) |
| 239 | 3 | 9 | 41 | 1 | 34 | 27 | 1 | 1 | 1 (0/1) |
| 240 | 2 | 3 | 45 | 3 | 11 | 2 | 5 | 4 | 1 (0/1) |
| 249 | 2 | 5 | 41 | 6 | 2 | 3 | 3 | 1 | 1 (0/1) |
| 252 | 28 | 2 | 49 | 3 | 8 | 28 | 5 | 1 | 1 (0/1) |
| 275 | 1 | 3 | 39 | 1 | 36 | 10 | 3 | 1 | 1 (1/0) |
| 276 | 6 | 3 | 48 | 4 | 4 | 5 | 1 | 4 | 2 (1/1) |
| 277 | 6 | 1 | 58 | 4 | 5 | 5 | 1 | 4 | 3 (1/2) |
| 278 | 3 | 2 | 44 | 3 | 1 | 11 | 1 | 3 | 2 (0/2) |
| 285 | 20 | 2 | 56 | 3 | 4 | 28 | 1 | 1 | 1 (0/1) |
| 334 | 2 | 2 | 41 | 1 | 2 | 5 | 27 | 1 | 1 (1/0) |
| 353 | **40** | 2 | 1 | 8 | 9 | 3 | 1 | 1 | 2 (2/0) |
| 354 | 1 | 9 | 43 | 1 | 2 | 5 | 3 | 10 | 4 (4/0) |
| 355 | 6 | 1 | 1 | 3 | 2 | 27 | 1 | 3 | 6 (4/2) |
| 356 | 37 | 3 | 39 | 1 | 2 | 19 | 11 | 1 | 1 (1/0) |
| 357 | 3 | 2 | 41 | 1 | 2 | 3 | 7 | 1 | 1 (1/0) |
| 358 | 3 | 5 | 50 | 1 | 2 | 1 | 3 | 1 | 1 (1/0) |
| 359 | 1 | 2 | 44 | 1 | 17 | 3 | 1 | 1 | 1 (1/0) |
| 360 | 3 | 5 | 50 | 1 | 4 | 5 | 1 | 1 | 1 (1/0) |
| 361 | 37 | 2 | 41 | 3 | 6 | 3 | 11 | 1 | 1 (1/0) |
| 362 | 3 | 2 | **64** | 1 | 2 | 5 | 3 | 1 | 1 (1/0) |
| 363 | 2 | 3 | 39 | 1 | 2 | 5 | 3 | 1 | 3 (3/0) |
| 364 | 37 | 2 | 1 | 1 | 36 | 27 | 1 | 1 | 1 (1/0) |
| 365 | 36 | 5 | 41 | 20 | 2 | 2 | 3 | 1 | 1 (1/0) |
| 366 | 16 | 5 | **65** | 29 | 7 | 10 | 1 | 1 | 2 (2/0) |
| 367 | 2 | 3 | **65** | 1 | 7 | 3 | 6 | 1 | 1 (1/0) |
| 368 | 7 | 2 | **66** | 1 | 2 | 4 | 1 | 3 | 1 (1/0) |
| 369 | 36 | 5 | 41 | 20 | 2 | 2 | 3 | 10 | 1 (1/0) |
| 370 | 3 | 2 | 50 | 10 | 2 | 5 | 3 | 1 | 1 (1/0) |
| 371 | 37 | 3 | 39 | 1 | 2 | 3 | 11 | 1 | 1 (1/0) |
| 372 | 14 | 2 | 53 | 1 | 2 | 3 | 1 | 1 | 2 (2/0) |
| 373 | 1 | 3 | 51 | 1 | 36 | 10 | 1 | 1 | 1 (1/0) |
| 374 | 25 | 2 | 1 | 1 | 9 | 10 | 6 | 1 | 1 (1/0) |
| 375 | 3 | 2 | 41 | 3 | 7 | 3 | 1 | 1 | 1 (1/0) |
| 376 | 1 | 3 | 39 | 13 | 36 | 3 | 3 | 1 | 1 (1/0) |
| 377 | 2 | 2 | 1 | 1 | 7 | 19 | 11 | 1 | 1 (0/1) |
| 378 | 1 | 3 | 44 | 28 | 2 | 3 | 12 | 1 | 1 (0/1) |
| 379 | 2 | 3 | 44 | **32** | 4 | 5 | **45** | 1 | 1 (0/1) |
| 380 | 37 | 2 | 55 | 3 | 2 | 7 | 1 | 1 | 3 (0/3) |
| 381 | 3 | 22 | 43 | 4 | 4 | 5 | 3 | 4 | 1 (0/1) |
| 382 | 28 | 2 | 49 | 9 | 3 | 28 | 5 | 1 | 2 (0/2) |
| 383 | 3 | 9 | 41 | 1 | 34 | 27 | 3 | 1 | 1 (0/1) |
| 384 | **40** | 3 | 50 | 1 | 7 | 2 | 11 | 1 | 1 (0/1) |
| 385 | **41** | 3 | 1 | 1 | 4 | 16 | 3 | 1 | 1 (0/1) |
| 386 | 3 | 5 | 50 | 1 | 4 | **33** | 3 | 1 | 2 (0/2) |
| 387 | 1 | 17 | 41 | 1 | 7 | 16 | 3 | 9 | 2 (0/2) |
| 388 | 3 | 3 | 39 | 1 | 4 | 3 | 3 | 1 | 2 (0/2) |
|  |  |  |  |  |  |  |  |  |  |
|  |  |  |  |  |  |  |  |  |  |
|  |  |  |  |  |  |  |  |  |  |
|  |  |  |  |  |  |  |  |  |  |
| ST | Allelic profile^a^ | | | | | | | | No. of strains detected  (*cnm*-positive/*cnm*-negative) |
|  | *tkt* | *glnA* | *gltA* | *glk* | *aroE* | *murI* | *lepC* | *gyrA* |  |
| 389 | 1 | 14 | **67** | 4 | 4 | 3 | 11 | 1 | 1 (0/1) |
| 390 | 37 | 16 | 60 | 13 | 2 | 18 | 22 | 15 | 2 (0/2) |
| 391 | 3 | 5 | 50 | 1 | 7 | **33** | 3 | 1 | 2 (0/2) |
| 392 | 4 | 1 | 1 | 2 | 2 | 2 | **46** | 1 | 1 (0/1) |
| 393 | 3 | 14 | 42 | 1 | 7 | 19 | 11 | 9 | 1 (0/1) |

ST, sequence type. ^a^ New alleles identified in this study are indicated in bold.
